# Oncologic Outcomes of Asian Men with Clinically Localized Prostate Cancer after Extraperitoneal Laparoscopic Radical Prostatectomy: A Single-Institution Experience

**DOI:** 10.1155/2011/748616

**Published:** 2010-12-19

**Authors:** Huai-Ching Tai, Ming-Kuen Lai, Chao-Yuan Huang, Shuo-Meng Wang, Kuo-How Huang, Shiu-Dong Chung, Shih-Chieh Jeff Chueh, Hong-Jeng Yu

**Affiliations:** ^1^Department of Urology, National Taiwan University Hospital and College of Medicine, No. 7 Chung-Shan S. Road, Taipei 100, Taiwan; ^2^Glickman Urological and Kidney Institute, Cleveland Clinic Foundation, 9500 Euclid Avenue, Cleveland, OH 44195, USA

## Abstract

*Purpose*. To evaluate the midterm oncologic results of extraperitoneal laparoscopic radical prostatectomy (EPLRP) for Asian men with localized prostate cancer. *Methods*. Between 2004 and 2009, 218 men underwent EPLRP at an Asian tertiary hospital. The mean preoperative prostate-specific antigen (PSA) was 15.5 ng/ml and mean Gleason score was 6.6. Stage distributions were cT1a-b in 21 cases, cT1c in 139, cT2 in 48 and cT3 in 10. Disease recurrence was defined as PSA ≥ 0.2 ng/mL in 2 consecutive measurements or initiation of secondary therapy. *Results*. Postoperative pathological stage was pT2a-b in 33 patients, pT2cN0 in 10, pT3a in 27, pT3b in 36, pT4 in 9 and pN1 in 10. Positive surgical margins occurred in 14.6% and 48.6% for pT2 and pT3 diseases, respectively (*P* < .001). The overall PSA recurrence-free survival at 3 and 5 years was 82.1% and 74.5%. By the pathological stages, 3-year recurrence-free survival was 92.4% (pT2), 81.1% (pT3a), 62.6% (pT3b-4) and 55.6% (pN1), respectively (*P* < .001). *Conclusions*. EPLRP is curative even for some locally advanced prostate cancers in a midterm follow-up. Even at an Asian center of low volume of radical prostatectomy EPLRP still provides oncologic outcomes similar to that of high volume centers.

## 1. Introduction

The incidence and mortality rate of prostate cancer in Taiwan, although not as high as those in the Western countries, were increasing significantly during the past decade [1]. The efficacy of various treatment modalities, especially for the potentially curable clinically localized prostate cancer, needs to be monitored carefully to ensure that the patients receive the best treatment available [2, 3]. Open retropubic radical prostatectomy (RRP) has been widely accepted for the treatment of choice for clinically localized prostate cancer since it is generally well tolerated with low morbidity and offers satisfactory oncologic and functional outcomes [4, 5]. For a minimally invasive approach to be accepted as a reasonable alternative to RRP, it must provide at least equivalent midterm oncologic and functional results and demonstrate relatively lower perioperative and long-term morbidities [6].

Laparoscopic radical prostatectomy (LRP) was first pioneered in 1992 by Schuessler et al. [7]. In 1997, they published the initial series of nine cases and concluded that LRP was feasible but not efficacious in comparison to the conventional counterpart [8]. However, after the modifications and standardization of the LRP technique by Guillonneau and Vallancien [9, 10] and Abbou et al. [11] in 1999, a progressively growing interest for LRP has blossomed among urologists. Since then, several studies comparing LRP with RRP have been reported in the literature [12–15]. However, there were limited reports from Taiwan and/or Asia, an area of relatively low-case volume of radical prostatectomy, to evaluate whether the potential advantages of such a technically demanding operation is really worthwhile.

We performed our first LRP in 2002 with a transperitoneal Montsouris approach [9] and introduced the extraperitoneal technique (EPLRP) since 2004 [16]. Until recently, we have completed almost 250 EPLRP procedures, and at least 180 cases have been followed up for more than one year at our institution. Herein, we reviewed the midterm oncologic outcomes of our EPLRP series and compared the results with those of other high-volume centers.

## 2. Methods

### 2.1. Patient Selection

Between December 2004 and June 2009, 218 consecutive men underwent EPLRP for clinically localized prostate cancers at National Taiwan University Hospital, a tertiary referral center. All procedures were performed by three urologic surgeons (SCC, MKL, and CYH). Men with concomitant operations other than total extraperitoneal (TEP) hernia repair were excluded from analysis.

### 2.2. Evaluation of Clinical Parameters

The preoperative clinical parameters including patient's age, preoperative prostate-specific antigen (PSA) level, biopsy Gleason score, and clinical stages were reviewed. The pathological data included Gleason score, pathological stages, positive surgical margin (PSM) rate, and presence of perineural invasion (PNI). PSM was considered if tumor was present at the inked margin of the specimen. Pathological staging was done using the 2002 AJCC TNM system and Gleason scoring was assigned according to modern convention. Recurrence of disease was defined as serum PSA value  ≥  0.2 ng/mL in at least two consecutive measurements or initiation of secondary therapy. Recurrence-free survival was defined as the time between EPLRP and the first PSA increase (≥0.2 ng/mL). Men who had a detectable postoperative PSA immediately after surgery or received adjuvant treatment (hormone or radiation) before an increasing postoperative PSA were assumed to have experienced failure at the time of surgery (i.e., recurrence-free survival  =  0 month). After EPLRP, all men except 13 received regular PSA monitoring and digital rectal examination (DRE) at our clinics every 3 months for the first 2 years and then every 6 months thereafter.

### 2.3. Surgical Procedures

To create the extraperitoneal working space for EPLRP procedure, we developed a novel technique with the assistance of a Visiport Optical Trocar [16]. After establishment of the working space with pneumo-extraperitoneum, LRP was executed by first controlling the deep dorsal vein complex and then dissecting the prostate and seminal vesicles by an antegrade approach. All patients received bilateral pelvic lymph node dissection (PLND, external iliac, and obturator fossa groups) despite preoperative risk stratification.

### 2.4. Statistical Analysis

The PSA recurrence-free survival was analyzed using the Kaplan-Meier method, and the log-rank test was used to compare survival curves of various subgroups. Survival curves were further stratified by preoperative PSA and pathological characteristics (Gleason score, pathological stages, PSM, and perineural invasion). The impact of various preoperative and postoperative risk factors on recurrence-free survival was analyzed using Cox regression analysis. A *P* value of less than  .05 was considered statistically significant. All analyses were performed with Statistical Package for the Social Science software (SPSS 13th ed., Chicago, IL, USA).

## 3. Results

### 3.1. General Characteristics

Preoperative patient characteristics are listed in Table 1. 

### 3.2. Pathological Results (Table 2)

Mean prostate volume was 43.7 gm (range 19 to 112). Pathological stage was pT2a-b in 33 (15.1%), pT2c in 103 (47.2), pT3a in 27 (12.4%), pT3b in 36 (16.5%), and pT4 in 9 (4.2%). All patients (100%) underwent bilateral PLND in external iliac and obturator fossa groups, which revealed positive lymph nodes in 10 cases (4.6%). Postoperative Gleason score was <7 in 52 (23.9%), 7 in 145 (66.5%), and >7 in 21 men (9.6%), respectively. PSM occurred in 27.1% of patients, including 20 of 137 of pT2 (14.6%), 35 of 72 of pT3 (48.6%), and 4 of 9 of pT4 (44.4%) disease.

### 3.3. Oncologic Outcomes (Table 2)

Thirteen patients have been lost during followup (5.9%). Seven patients (3.2%) had neoadjuvant treatment, and a total of 14 patients (6.4%) received adjuvant therapy before postoperative PSA started to increase, including external beam radiation alone in 3, hormone therapy alone in 7, and a combination of external beam radiation and hormone therapy in 4. 

None of the patients died of prostate cancer progression during the followup period. Three patients died of other causes, namely, acute myeloid leukemia, acute myocardial infarction, and sepsis in each one.

**Figure 1:**
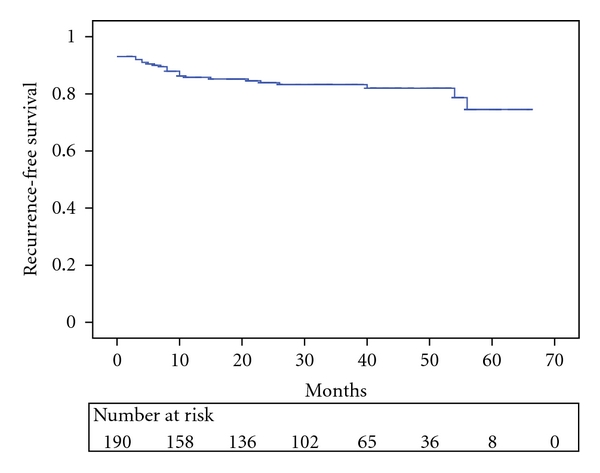
PSA recurrence-free survival after EPLRP in Taiwanese men.

During a median followup of 34 months (range 2 to 66), PSA recurrence occurred in 25 patients (13.1%), and we were only able to determine the location of recurrence in three patients (2 local and 1 metastatic). Of whom, 21 received secondary therapy including external beam radiation in 7, hormone therapy in 7, and a combination of radiation and hormone therapy in 7. The median time to progression was 31 months, with 33 months for pT2, 32.5 months for pT3a, 22 months for pT3b-4, and 11 months for pT2-4N1 disease. The actuarial overall PSA recurrence-free survival rate at 3 and 5 years was 82.1% and 74.5%, respectively (Figure 1). When stratified by pathological stages, the 3-year recurrence-free survival rate was 92.4% for pT2, 81.1% for pT3a, 62.6% for pT3b-4, and 55.6% for pN1 (*P* < .001, Figure 3).

**Figure 2:**
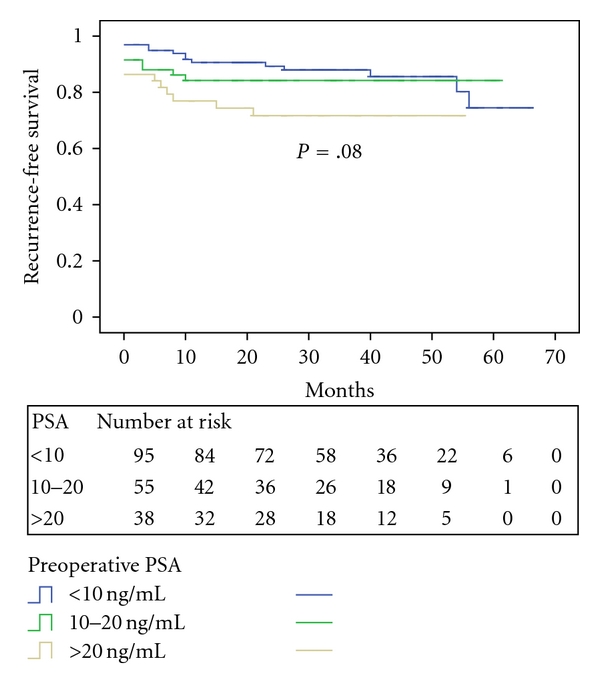
PSA recurrence-free survival stratified by preoperative PSA level.

**Figure 3:**
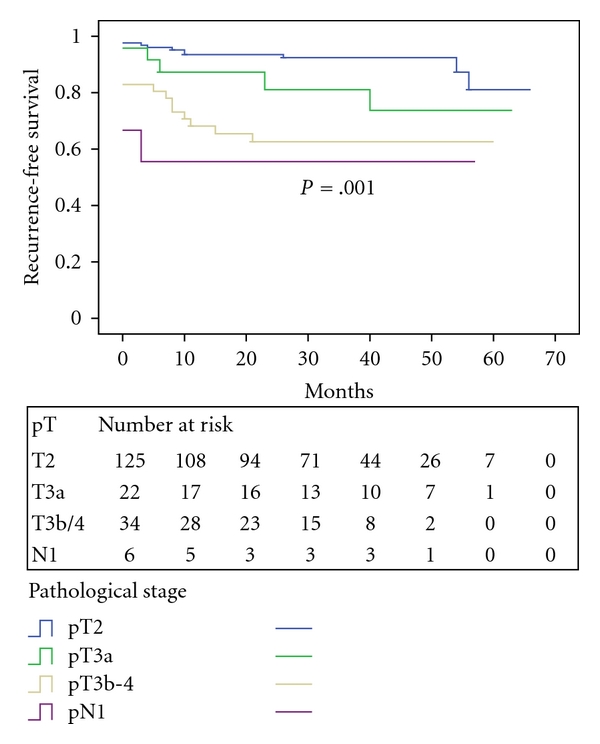
PSA recurrence-free survival stratified by pathological stage.

The recurrence-free survival by preoperative PSA strata is shown in Figure 2. The 3-year recurrence-free survival rate was 87.8% for PSA < 10 ng/mL, 84.3% for PSA 10–20 ng/mL, and 71.7% for PSA ≥ 20 ng/mL (*P* = .08).

**Figure 4:**
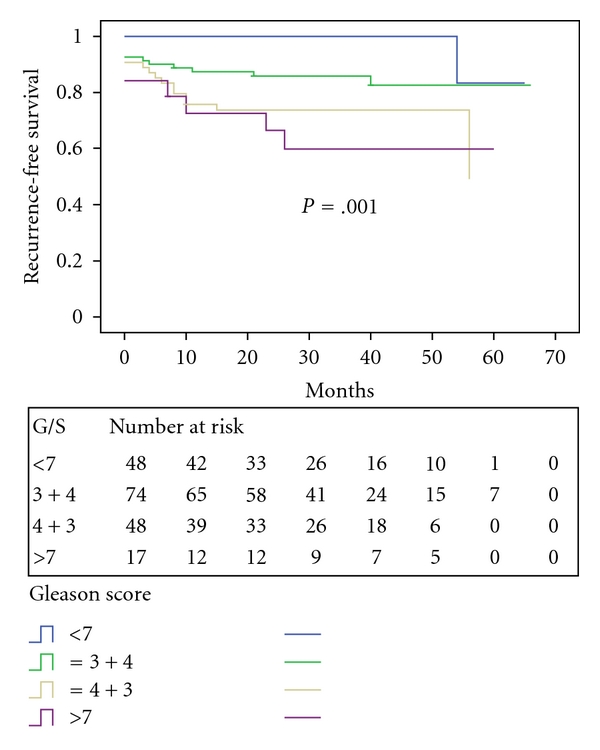
PSA recurrence-free survival stratified by Gleason score.

To study the prognostic value of Gleason score, we divided the patients into those with Gleason score < 7, score = 7, and score > 7. The 3-year recurrence-free survival was 83.3% for Gleason score < 7, 79.1% for score = 7, and 59.8% for score > 7 (*P* = .001, Figure 4). For patients with Gleason score 7, the 3-year recurrence-free survival was 73.7% and 85.8% for those with predominant grade 4 (i.e., 4 + 3) and grade 3 (i.e., 3 + 4) disease, respectively (*P* = .07). 

**Figure 5:**
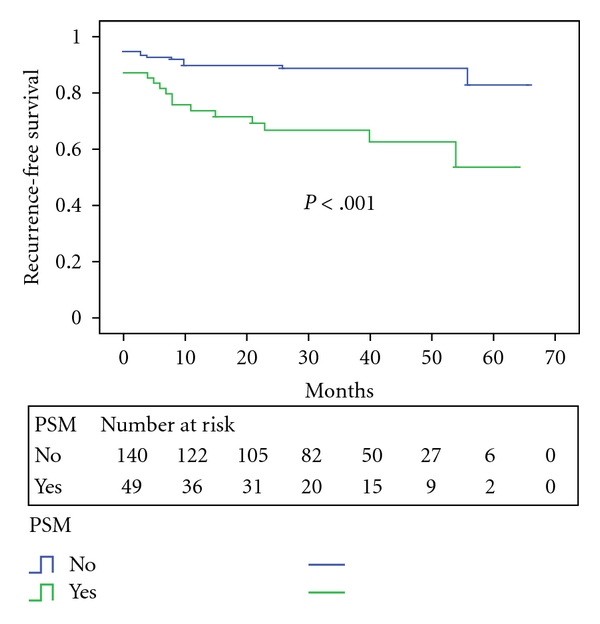
PSA recurrence-free survival stratified by surgical margin status.

**Figure 6:**
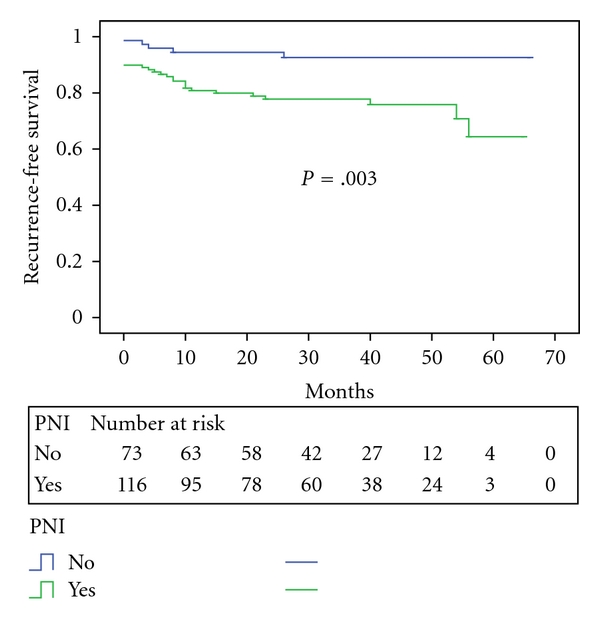
PSA recurrence-free survival stratified by the presence of perineural invasion (PNI).

 Men with PSM had a worse PSA recurrence-free survival rate of 66.8% at 3 years compared with 89.2% for those with negative surgical margins (*P* < .001, Figure 5). Recurrence-free Kaplan-Meier survival curves also revealed that presence of perineural invasion was also a significant parameter with regard to outcome (*P* = .003, Figure 6). 

While faily acceptable oncologic outcomes were achieved in our series, the functional outcomes of our EPLRP series showed that the percentages of the patients dry (use less than 1 pad/day) after surgery were 66% at 3 months after surgery, 92% at 6 months, and 96% at 18 months. Since not all these patients were done to preserve their neurovascular bundles and not all the patients have pre- and postoperative erection functions scores to be compared, we are not able to provide the erection functional outcome among this patient cohort.

## 4. Discussions

Radical prostatectomy is the major curative treatment for localized prostate cancer, and the open retropubic approach remains currently the gold standard option for such disease [21]. Published reports have shown that, in the hands of an experienced urological surgeon, this procedure is associated with minimal intraoperative and postoperative morbidity, without compromising the oncologic and functional outcomes [22]. Thus, it sets a high standard for the emerging technique, laparoscopic radical prostatectomy, to be an ideal alternative.

Several large series have addressed the oncologic effectiveness of LRP, with the PSM rates between 6.2% and 27.5% for pT2 tumors and between 31.1% and 68.0% for pT3 tumors. The 3-year actuarial recurrence-free survival rates were between 72% and 95% (between 89% and 98% for pT2 tumors and between 51% and 79% for pT3 tumors when stratified by pathological stage) [18, 23–26]. Moreover, Touijer et al. have even reported a longer oncologic result with 78% and 71% for overall 5-year and 8-year probability of freedom from recurrence, respectively [27]. However, all of these LRP data were predominantly based on the transperitoneal approach; information regarding the oncologic followup of EPLRP was limited (Table 3). Stolzenburg et al. reported the largest EPLRP series with 2400 cases, and the PSM rate was 8% for pT2 and 35.6% for pT3 cancers [20]. Paul et al. first published the midterm results of 1115 EPLRP procedures with a median followup of 35.6 months (range 1–92). The overall PSA recurrence-free survival rates were 84% at 3 years and 83% at 5 years. According to the pathological stages, 5-year progression-free survival rate was 93.4% for pT2 tumors, 70.2% for pT3 tumors, and 42.7% for pT4 tumors. Preoperative PSA, Gleason score, tumor stage, nodal status, and surgical margins were significant independent predictors of biochemical recurrence-free survival [19].

To our knowledge, the present study was the only series describing the oncologic results of EPLRP from Asia, an area of relatively low incidence of prostate cancer and low volume of radical prostatectomy. Compared to other cohorts, patients in the present study seemed to have a higher preoperative PSA level and more unfavorable pathological stages (pT3-pN1) after surgery. However, our findings still provide acceptable 3-year and 5-year PSA recurrence-free survival in 82.1% and 74.5% of patients, respectively. When further classified by pathological stages, our EPLRP recurrence-free survival rate at 3 years was 92.4% for pT2, 81.1% for pT3a, 62.6% for pT3b-4, and 55.6% for pT1-4N1 cancers. The overall PSM rate was 27.1%, with 14.6% for pT2 and 47.9% for pT3 disease. Comparison of pathological and oncologic outcomes with other published EPLRP series is listed in Table 3. All these parameters of cancer control rates were within the confidence ranges reported by other high-volume centers using either open retropubic or laparoscopic surgery. Thus, we suggest that, even in an area of low prostate cancer incidence, LRP performed with extraperitoneal approach for patients with clinically localized prostate cancer is an oncologically safe and effective treatment.

Although the case number in the current series was not enormous, we observed not only comparable oncologic outcomes for pT2 prostate cancer patients but also acceptable 3-year oncologic outcomes even for patients with advanced localized prostate cancer (pT3 and pT4). This coincides with the recent concept that radical prostatectomy might provide cure for advanced localized prostate cancer and thus should be offered as a valuable and viable treatment alternative when consulting such patients preoperatively [28, 29].

In a multivariate Cox model, we found some risk factors ominous for PSA recurrence-free survival for our patient population, namely, higher preoperative PSA level, higher pathological stage (≥pT3), higher Gleason score (>7), existence of positive surgical margins, or/and perineural invasion on the pathological analysis (Table 4). These risk factors were similar to those previously reported in other series [20, 23–25, 30].

We performed our first LRP in 2002 with a transperitoneal approach. After initial successful experience in 30 cases, we started to perform the extraperitoneal technique since 2004 with the original idea of its potential advantage in preserving peritoneal integrity. This single-institution EPLRP experience is herein presented to demonstrate that EPLRP is a satisfactory and viable option in treating patients with clinically localized prostate cancer.

## 5. Conclusions

Based on our midterm followup data, EPLRP provided 3- and 5-year PSA recurrence-free survival in 82.1% and 74.5% of patients, respectively. We suggest that, even in an area of low prostate cancer incidence, LRP performed with extraperitoneal approach is an oncologically safe and effective treatment for clinically localized prostate cancer. Long-term oncologic data are needed to confirm the midterm results.

## Figures and Tables

**Table 1:** Clinical patient and pathological characteristics.

No. patients	218
Mean age at surgery (year, range)	64.6 (45–82)
Mean preop. PSA (ng/mL, range)	15.5 (0.26–185)
Preop. PSA (ng/mL)	
<4	7 (3.2%)
4–10	96 (44.0%)
10–20	68 (31.2%)
>20	47 (21.6%)
Clinical stage (%)	
cT1a-b	21 (9.6%)
cT1c	139 (63.8%)
cT2	48 (22.0%)
cT3	10 (4.6%)
Mean biopsy Gleason score (range)	6.6 (3–10)
Biopsy Gleason score (%)	
<7	99 (45.4%)
= 7	93 (42.7%)
>7	26 (11.9%)

**Table 2:** Pathological and oncologic outcomes.

Median followup (months)	34 (2–66)
Pathological stage (%)	
pT2a-bN0	33 (15.1%)
pT2cN0	103 (47.2%)
pT3aN0	27 (12.4%)
pT3bN0	36 (16.5%)
pT4N0	9 (4.2%)
pT1-4N1	10 (4.6%)
Pathological Gleason sum (%)	
<7	52 (23.9%)
= 7	145 (66.5%)
3 + 4	87
4 + 3	58
>7	21 (9.6%)
Positive surgical margins (%)	
Overall	59 (27.1%)
pT2	20 (14.6%)
pT3	35 (48.6%)
pT4	4 (44.4%)
Perineural invasion (%)	
Overall	136 (62.4%)
pT2	64 (47.1%)
pT3/4	62 (45.6%)
pN1	10 (7.3%)
PSA recurrence (%)	
Overall	25 (13.1%)
pT2	10 (7.3%)
pT3a	4 (14.8%)
pT3b	9 (25.0%)
pT1-4N1	2 (20.0%)

**Table 3:** Oncologic outcomes of extraperitoneal laparoscopic radical prostatectomy (EPLRP) series.

	Rozet et al. [17]	Rassweiler et al. [18]	Paul et al. [19]	Stolzenburg et al. [20]	Tai et al. [16]
Study period	1998–2004	1999–2004	2000–2007	2001–2008	2004–2009
No. institutions	1	1	1	3	1
No. surgeons	4	—	3	9	3
No. patients	600	500	1115	2400	218
Mean age (yrs)	62 (47–73)	64.0 (43–81)	62.5 (42–81)	63.3 (41–81)	64.6 (45–82)
Median followup (months)	12	40	35.6	—	34
Mean preoperative PSA (ng/mL)	7.4	11.7 (0.08–93)	9.8 (0.8–99)	9.8 (0.08–93)	16.3 (0.7–185)
Pelvic lymph node dissection (%)	—	83.4%	41.6%	50.8%	100%
Pathological stage					
pT2	72.0%	59.2%	59.5%	70.5%	62.3%
pT3a	19.2%	21.4%	22.4%	19.7%	12.4%
pT3b	8.8%	12.8%	9%	9.4%	16.5%
pT4	—	3.8%	6.9%	0.3%	4.2%
pT1-4N1	—	1.4%	2.2%	—	4.6%
Positive surgical margin (%)					
Overall	17.7%	19.0%	26.0%	16.0%	27.1%
pT2	14.6%	7.4%	16.1%	8.1%	14.6%
pT3	25.6%	31.8%	34.6%	35.7%	47.9%
PSA recurrence-free survival rate					
3-year	—	83.0%	84%	—	82.1%
5-year	—	73.1%	83%	—	74.5%

**Table 4:** Multivariate Cox proportional hazard model for prediction of PSA recurrence.

	HR	95% CI	*P* value
Preoperative PSA level	1.501	1.005, 2.243	.047
Pathological stage	1.543	1.281, 1.860	<.001
Gleason score	2.828	1.564, 5.112	.001
Surgical margin	2.162	1.477, 3.165	<.001
Perineural invasion	3.765	1.460, 9.710	.006
